# Causal relationship between gut microbiota and idiopathic pulmonary fibrosis: A two-sample Mendelian randomization

**DOI:** 10.1097/MD.0000000000039013

**Published:** 2024-07-19

**Authors:** Shiqin Fan, Baorui Xue, Jing Ma

**Affiliations:** a Department of Intensive Care Medicine, Liyuan Hospital, Tongji Medical College of Huazhong University of Science and Technology, Wuhan, Hubei, China; b Department of Endocrinology, Liyuan Hospital, Tongji Medical College, Huazhong University of Science and Technology, Wuhan, Hubei, China.

**Keywords:** gut microbiota (GM), idiopathic pulmonary fibrosis (IPF), Mendelian randomization (MR)

## Abstract

To explore the causal relationship between gut microbiota (GM) and Idiopathic pulmonary fibrosis (IPF), we performed a two-sample Mendelian randomization (MR). GM was used as an exposure factor, and instrumental variables were determined from the GWAS of 18,340 participants. GWAS of IPF (including 1028 IPF patients and 196,986 controls) from the FinnGen was used as the outcome factor. The primary analysis method is the inverse variance weighted (IVW) method, and sensitivity analysis was used to validate the reliability. Family Bacteroidaceae (OR = 1.917 95% CI = 1.083–3.393, *P* = .026), order Gastranaerophilales (OR = 1.441 95% CI = 1.019–2.036, *P* = .039), genus Senegalimassilia (OR = 2.28 95% CI = 1.174–4.427, *P* = .015), phylum Cyanobacteria (OR = 1.631 95% CI = 1.035–2.571, *P* = .035) were positively correlated with IPF. FamilyXIII(OR = 0.452 95% CI = 0.249–0.82, *P* = .009), order Selenomonadale (OR = 0.563 95% CI = 0.337–0.941, *P* = .029), genus Veillonella (OR = 0.546 95% CI = 0.304–0.982, *P* = .043) (OR = 0.717 95% CI = 0.527–0.976, *P* = .034), genus Ruminococcusgnavus (OR = 0.717 95% CI = 0.527–0.976, *P* = .034), genus Oscillibacter (OR = 0.571 95% CI = 0.405–0.806, *P* = .001) was negatively correlated with IPF. Sensitivity analysis showed no evidence of pleiotropy or heterogeneity (*P* > .05). The results of MR demonstrated a causal relationship between GM and IPF. Further studies are needed to investigate the intrinsic mechanisms of the GM in the pathogenesis of IPF.

## 1. Introduction

Idiopathic pulmonary fibrosis (IPF), characterized by impaired lung function and persistent scarring of lung tissue, is a life-threatening lung disease.^[1]^ Studies have shown that the incidence of IPF has been rising over the last decade, resulting in a heavy healthcare burden.^[2,3]^ Repeated lung microinjuries may ultimately result in lung scarring, which subsequently leads to pulmonary fibrosis gradually.^[4]^ Eventually, these processes lead to difficulties in breathing and a dramatic decline in lung function. To date, the exact etiology and pathogenesis of IPF have not been determined. Therefore, early identification of potential risk factors can effectively reduce the mortality rate of IFP, which will help in the early prevention of IFP and provide a rational treatment plan.

The gut microbiota (GM) presented in the gastrointestinal tract helps to maintain normal physiological processes and keeps our body in a healthy condition.^[5]^ The GM maintains the barrier function of the intestinal mucosal epithelium, thus preventing the spread of bacteria to distant infections.^[6]^ The recent study found that there has been a link between GM and lung disease. The GM can stimulate an immune response, which in turn may impact the lungs. This connection between the gastrointestinal tract and the lungs is described as the gut-lung axis.^[7]^ Several studies have demonstrated that GM may have an impact on several lung illnesses, including viral pneumonia,^[8]^ acute lung injury,^[9]^ pulmonary arterial hypertension,^[10]^ chronic obstructive pulmonary disease,^[11]^ lung cancer^[12]^ and IPF.^[13]^ Metabolites produced by the GM, that is, amino acids, short-chain fatty acids and bile acids, may contribute to the development of pulmonary fibrosis by immunomodulating, accumulating extracellular matrix, and affecting energy metabolism.^[14]^ These metabolites have strong anti-inflammatory effects and maintain the intestinal mucosal barrier by promoting the production of intestinal mucins and preventing bacterial translocation.^[15]^ Schuijt et al reported that alveolar macrophages were more resistant to Streptococcus pneumoniae after treating genetically modified-deficient mice with fecal transplants,^[16]^ suggesting that the GM provided a protective effect on the lungs.

In conclusion, the causal association between GM and IPF has not been identified. Therefore, further investigations of the potential causal relationship between GM and IPF are crucial. We explored the potential causal association between GM and IPF using Mendelian randomization (MR), a statistical strategy based on genome-wide association analysis that excludes the influence of confounding factors. Our study was to explore the link between GM and IPF and provide feasible approaches for individualized treatment of IPF.

## 2. Methods and materials

### 2.1. Overall study design

**Figure 1.**
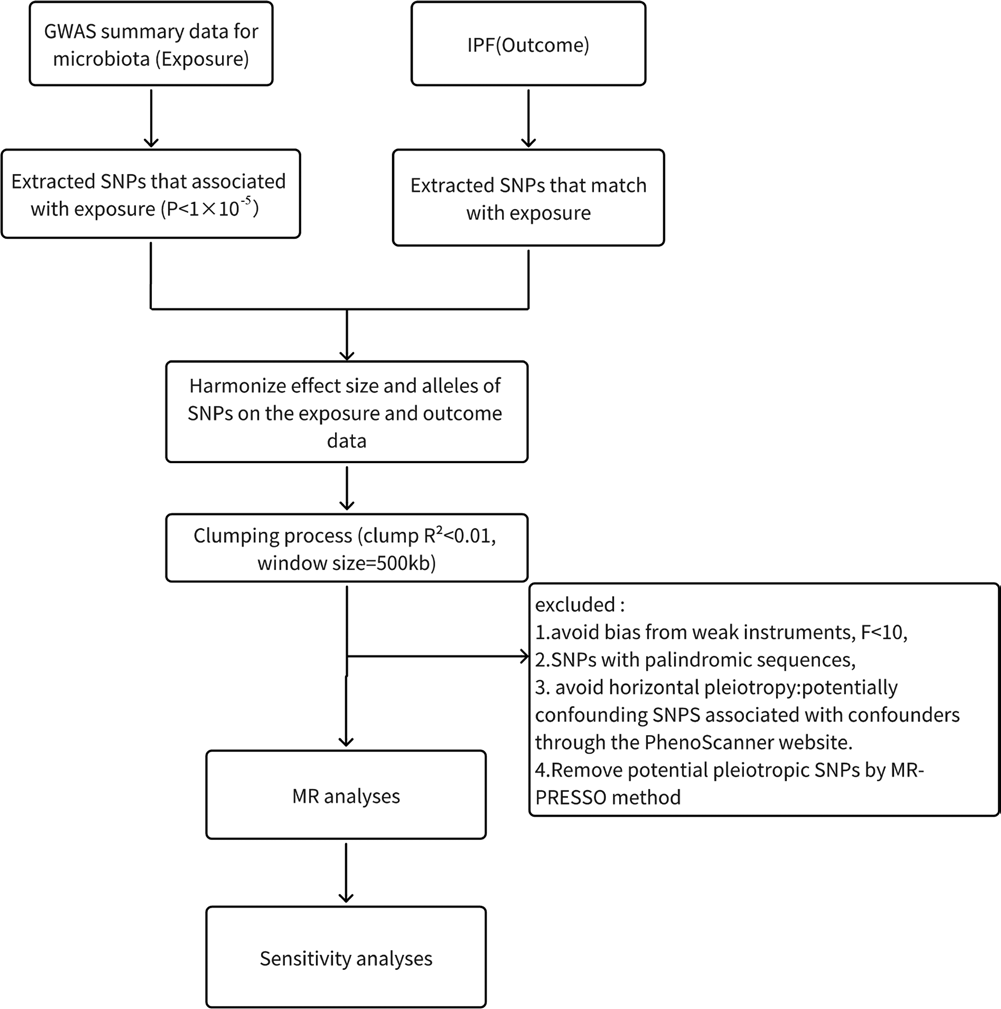
The flowchart of the study.

**Figure 2.**
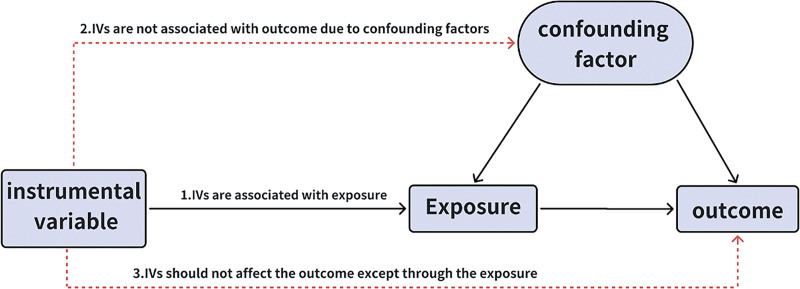
An overview of the study design.

An overview of our study is shown in Figure 1. We evaluated the causal relationship between the GM and IPF using a 2-sample MR method. Our 2-sample MR study builds on the following 3 main assumptions: correlation assumption: Genetic variants selected as instrumental variables (IVs) were related to exposure (GM); assumption of independence: IVs cannot affect the outcome through confounders factors; and exclusion restriction assumption: the IVs could only influence outcomes through exposure and not in other ways (Fig. 2).

### 2.2. Data sources (exposure and outcome data)

The complete GM GWAS data were obtained from the MiBioGen (www.mibiogen.org), an international consortium. The consortium investigated the impact of human genetics on the GM and conducted a multi-ethnic GWAS study. It recorded 18,340 participants (24 cohorts) from 11 European countries, incorporating a total of 211 taxonomic units (131 genera, 35 families, 20 orders, 16 phylums, and 9 orders).^[17]^ Summary statistics for IPF data were obtained from FinnGen version 9 (https://r9.risteys.finngen.fi/, accessed on November 12, 2023) (Table 1).

**Table 1:** Characteristics of GWASs for gut microbiota traits and IPF.

Trait	Sample size	Consortium	Link	Yr
Gut microbiota	18,340	MiBioGen	www.mibiogen.org	2021
IPF	1028 patients 196,986 controls	FinnGen	https://r9.risteys.finngen.fi/	2021

GWAS = genome-wide association studies, IPF = idiopathic pulmonary fibrosis, SNP = single-nucleotide polymorphism.

### 2.3. Selection of IVs

GM was used as the exposure agent, while IPF was used as the outcome.

To ensure that our conclusions are accurate and reliable, we conducted strict quality control of single-nucleotide polymorphisms (SNPs). First, to obtain a more comprehensive set of SNPs associated with the GM, we chose a locus-wide significance (*P* < 1 × 10^-5^).^[18]^ Second, a linkage disequilibrium analysis (R^2^ < 0.01, clustering distance = 500 kb) was conducted to ensure statistical independence. Third, to avoid bias from weak IVs, we assessed the effectiveness of the IVs with the formula F = β^2^_exposure_/SE^2^_exposure_ and excluded IVs with F < 10.^[19]^ Fourth, we excluded SNPs with palindromic sequences (e.g., G/C or A/T alleles) to improve the accuracy of the analysis. Fifth, PhenoScanner (www.phenoscanner.medschl.cam.ac.uk) was then used to minimize the correlation of SNPs with confounders,^[20]^ such as BMI, smoking and type 2 diabetes.

### 2.4. MR analysis

Our MR analysis consisted of 5 methods (inverse variance weighted [IVW], MR Egger, weighted median [WM], simple model and weighted model), and IVW was chosen as the major approach of analysis. The WM and MR Egger were applied to validate the robustness of IVW estimates, which can supplement the IVW method and provide wider confidence intervals.^[21]^ The estimates are expressed as odds ratios (OR) and 95% confidence intervals (CI), with *P* < .05 showing statistical significance.^[22]^ We further visualized the results of GM and IPF causality as results to facilitate interpretation.

### 2.5. Sensitivity analysis

We evaluated heterogeneity using Cochran Q test (*P* < .05 indicates significant heterogeneity, in which case we used a random effects IVW model; conversely, we used a fixed effects IVW model).^[23]^ The pleiotropic analysis was preliminarily judged by the intercept of MR–Egger regression and MR-PRESSO global. We used leave-one-out tests^[24]^ to rule out the effect of individual SNPs on the causal relationship between GM taxa and IPF, with the aim of further determining the robustness of the MR results.

### 2.6. Statistical analysis

Our statistical analyses were conducted with the “Two Sample MR” (version 0.5.6) package in R software (version 4.2.1). We believe that *P* < .05 represents statistical significance according to the IVW methods.

## 3. Results

### 3.1. Selection of IVs

Among the included IVs, phenoscanner revealed no potential IVs related to other risk factors. Our calculated F-statistics for IVs ranged between 17.68 and 30.06, all of which were >10. This means that the presence of weak IVs is less likely, and the chosen SNPs had a strong effect on IVs. All SNPs were characterized as shown in supplementary table 1. http://links.lww.com/MD/N240

### 3.2. MR analysis

**Figure 3.**
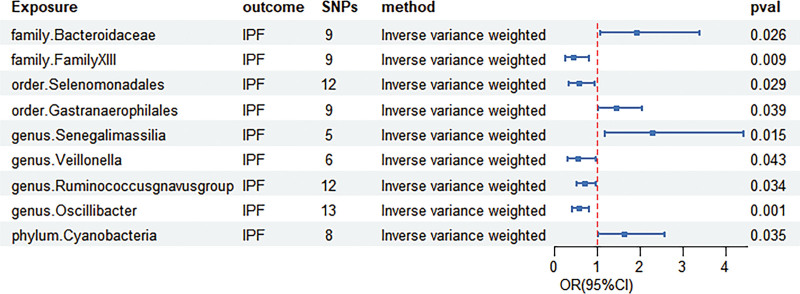
Forest plots.

**Figure 4.**
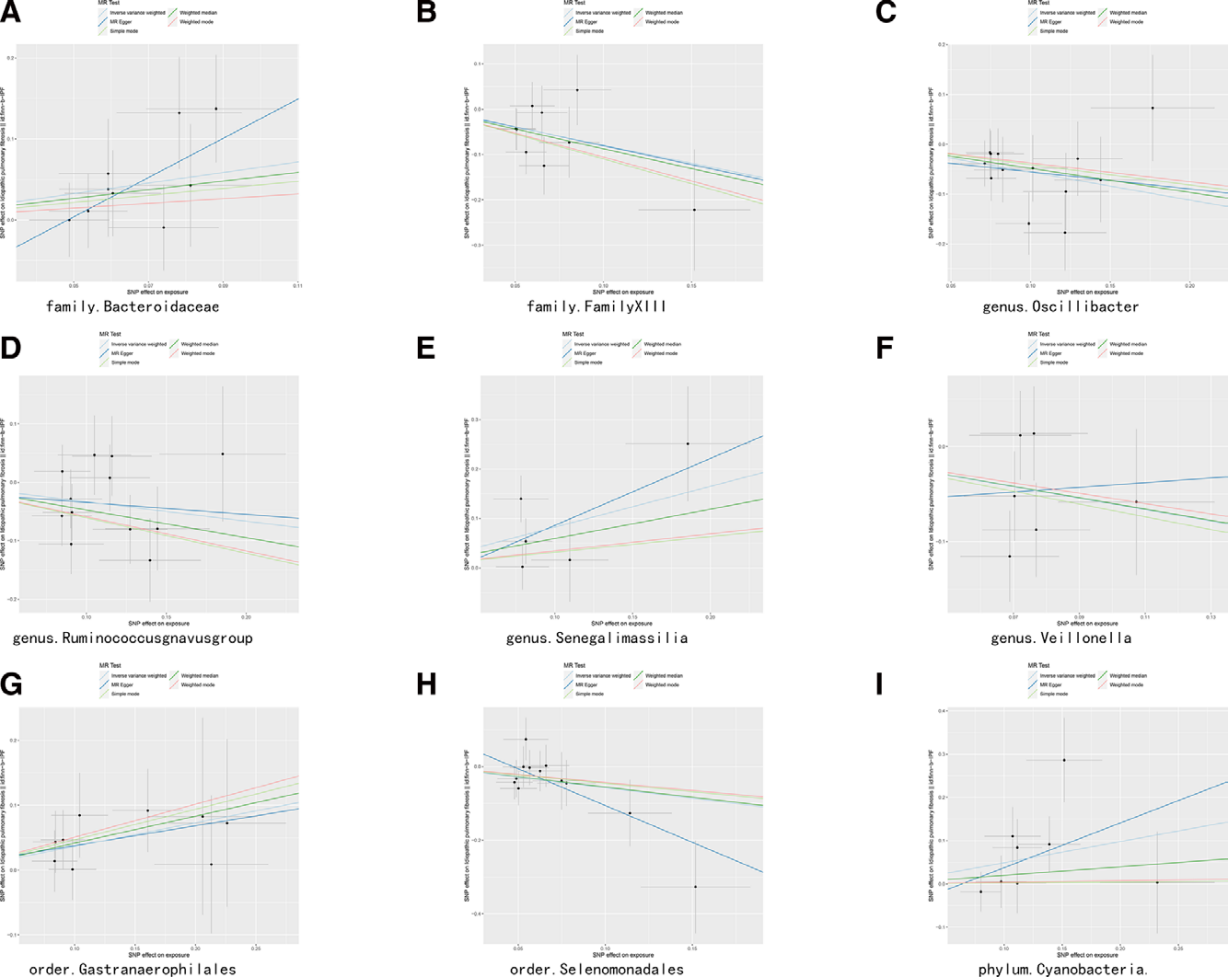
Scatter plots of this study.

IVW identified Gastranaeroph ‡table_number ‡table_numberilales (OR = 1.441 95% CI = 1.019–2.036, *P* = .039), Bacteroidaceae (OR = 1.917 95% CI = 1.083–3.393, *P* = .026), Senegalimassilia (OR = 2.28 95% CI = 1.174–4.427, *P* = .015), and Cyanobacteria (OR = 1.631 95% CI = 1.035–2.571, *P* = .035) as predisposing factors for IPF. In contrast, FamilyXIII (OR = 0.452 95% CI = 0.249–0.82, *P* = .009), Selenomonadale (OR = 0.563 95% CI = 0.337–0.941, *P* = .029), Veillonella (OR = 0.546 95% CI = 0.304–0.982, *P* = .043), Ruminococcusgnavus (OR = 0.717 95% CI = 0.527–0.976, *P* = .034), and Oscillibacter (OR = 0.571 95% CI = 0.405–0.806, *P* = .001) were protective factors for IPF (supplementary table 2) http://links.lww.com/MD/N240. The causal effect between GM and IPF is shown in a forest plot (Fig. 3), and the results of MR are presented in a scatter plot (Fig. 4).

### 3.3. Sensitivity analysis

**Figure 5.**
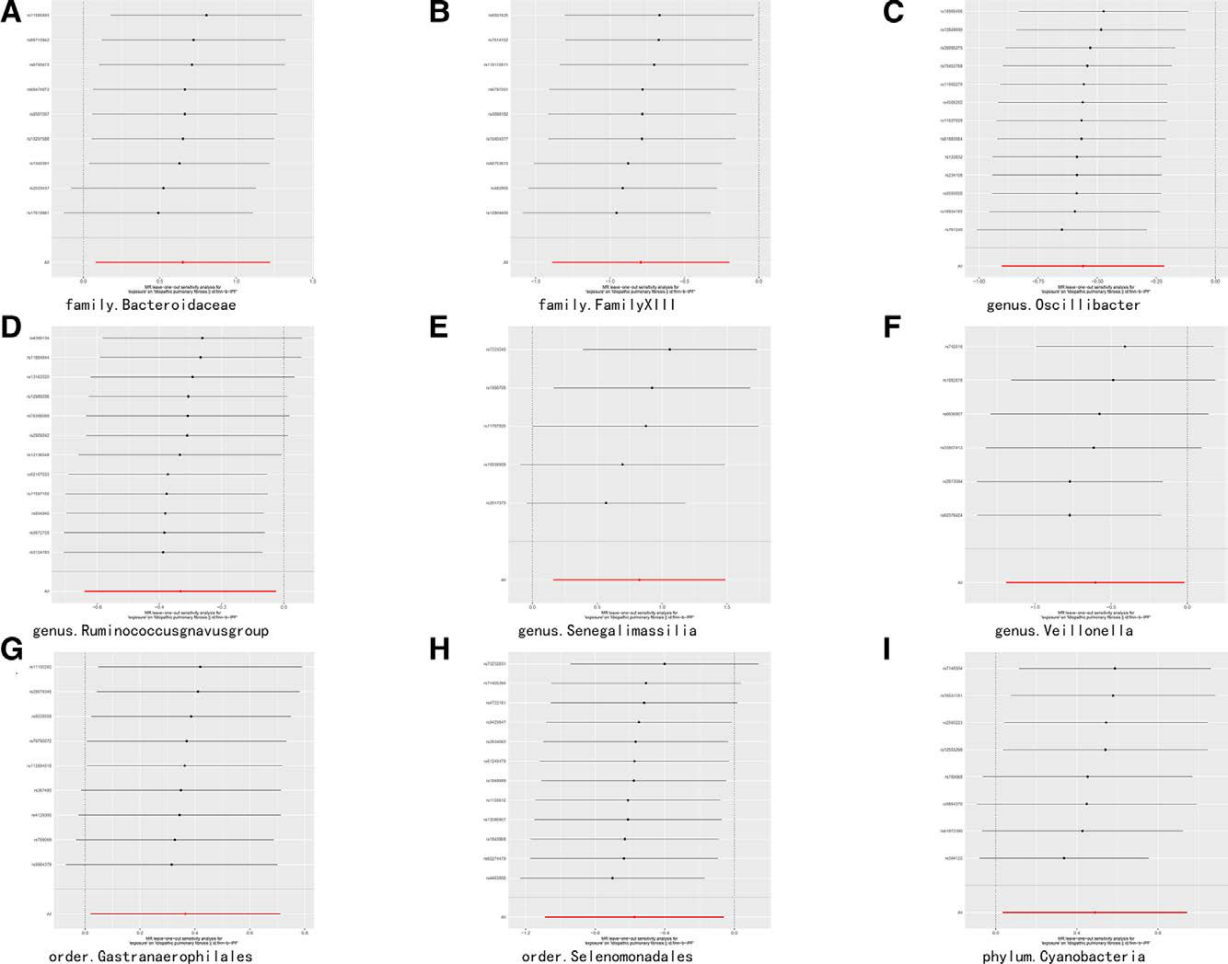
Leave-one-out plots.

The sensitivity analysis is presented in supplementary table 2. http://links.lww.com/MD/N240. The results of MR Egger and IVW analyses revealed the following: Bacteroidaceae (IVW: *P* = .77, MR Egger: *P* = .84); Family XIII (IVW: *P* = .58, MR Egger: *P* = .47); Oscillibacter (IVW: *P* = .71, MR Egger: *P* = .64); Ruminococcusgnavusgroup (IVW: *P* = .50, MR Egger: *P* = .41); Senegalimassilia (IVW: *P* = .20, MR Egger: *P* = .13); Veillonella (IVW: *P* = .31, MR Egger: *P* = .21); Gastranaerophilales (IVW: *P* = .98, MR Egger: *P* = .99); and Selenomonadales (IVW: *P* = .59, MR Egger: *P* = .76). Cyanobacteria (IVW: *P* = .17, MR Egger: *P* = .12) had no heterogeneity (Table 2). The results of the MR-PRESSO global test showed no horizontal pleiotropy (MR-PRESSO-*P* > .05). In addition, “leave-one-out” showed that the outcome was not impacted by removal of each SNP, providing robust evidence for the MR results (Fig. 5). It is worth noting that the slope of the IVW method in the MR results for the genus Veillonella has the opposite effect to that of the MR Egger method. However, we did not find horizontal pleiotropy according to the MR-PRESSO global test, suggesting that there is some kind of underlying pleiotropy subtly influencing our results.

**Table 2:** Sensitivity analyses of causality between GM taxa and IPF based on MR results.

Bacterial taxa	Q-P (IVW)	Q-P (MR Egger)	MR-PRESSO-P	Pleiotropy- P
family Bacteroidaceae	0.77	0.84	0.74	0.27
family FamilyXIII	0.58	0.47	0.64	0.97
order Selenomonadales	0.59	0.76	0.62	0.13
order Gastranaerophilales	0.98	0.99	0.98	0.91
genus Senegalimassilia	0.20	0.13	0.29	0.74
genus Veillonella	0.31	0.21	0.47	0.76
genus Ruminococcusgnavus	0.50	0.41	0.51	0.86
genus Oscillibacter	0.71	0.64	0.72	0.74
Phylum Cyanobacteria	0.17	0.12	0.16	0.77

## 4. Discussion

We conducted a 2-sample MR analysis using published GWAS statistics to investigate the causal relationship between the GM and IPF. These results suggest that several GM may contribute to or prevent the development of IPF. Our study provides fairly strong genetic evidence that the occurrence of IPF was associated with a greater abundance of Bacteroidaceae, Gastranaerophilales, Senegalimassilia, and Cyanobacteria. In contrast, we observed that higher levels of Family XIII, Selenomonadale, Veillonella, Ruminococcusgnavus, and Oscillibacter were associated with lower IPF risk. Therefore, purposefully aiming to modulate the abundance of the GM may influence the progression of IPF.

Although the exact mechanisms of action of GM in the pathogenesis of IPF are unknown, there are several discoveries demonstrating that GM plays an important role in the development of IPF. GM could break down carbohydrates to produce short-chain fatty acids (SCFA),^[25]^ which can reduce the generation of proinflammatory cytokines, including interleukin-6 and IL-1β, and thus affect lung immunomodulation.^[26]^ The 2 primary SCFAs butyric acid and propionic acid may promote barrier function in the respiratory epithelial mucosa. In an experiment involving mice with pulmonary fibrosis, it was found that treatment of mice with butyric acid reduced pulmonary fibrosis,^[27]^ indicating that butyric acid may alleviate the severity of IPF.^[28]^ Additionally, pulmonary fibrosis is modulated by amino acids generated by GM metabolism. For example, glutamate contributes to the formation of pulmonary fibrosis through collagen deposition and resistance to apoptosis.^[27]^ Arginine, on the other hand, blocks the progression of pulmonary fibrosis with immune modulation and by reducing the contribution of fibroblasts.^[29]^ Additionally, a rat experiment revealed that bile acids can induce pulmonary fibrosis through several mechanisms.^[30]^

Gastranaerophilales have been found to promote the production of plasma lipopolysaccharides (LPS),^[31]^ which invade the circulation via intestinal lesions and cause inflammatory reactions and cardiovascular diseases.^[32]^

SCFA can reduce inflammation induced by LPS, thus preserving the integrity of the gut mucosal barrier.^[33]^ However, Gastranaerophilales can lead to a decrease on SCFAs in the feces,^[31]^ which are associated with the physiological function of the lungs. This is consistent with our findings that Gastranaerophilales increases the risk of IPF. The Bactroidaceae is a family of bacteria in the order Bacteroidales, the type genus for this family is Bacteroides. It has been found that dietary fiber could impact GM to exert different biological effects by altering the ratio of Firmicutes to Bacteroidetes. Metabolites produced by the GM activate macrophages and dendritic cells to exert their biological effects in the lungs, ultimately exacerbating lung inflammation and altering the immune environment of the lungs.^[34,35]^

Bacteroides stimulate the release of proinflammatory cytokines (e.g., IL-1, CXCL8, IFN-γ, and tumor necrosis factor-α), which can lead to a severe inflammatory response.^[36]^ Bacteroidetes can increase bile acid levels in the liver through 2 pathways: catabolizing bile acids by bile salt hydrolases and causing a decrease in the absorption of bile acids in the ileum.^[37]^ Bile acids can induce the formation of IPF, which is consistent with our results that Bacteroidaceae is positively related to the development of IPF. Senegalimassilia has been found to be more abundant in cirrhotic patients with exacerbated steatosis.^[38]^ The gut-liver-lung axis was first identified and proposed in 1991. The GM can invade through the disrupted intestinal mucosal barrier, then enter the liver via the portal vein and release inflammatory mediators, which bind to lung endothelial cells and cause lung damage.^[39]^ Cyanobacteria have been found to promote the progression of hepatocellular carcinoma by releasing toxins into the liver and may also cause disorders of lipid metabolism, which may affect hepatocellular carcinoma.^[40]^ Both of these bacteria aggravate liver disease, but no study has confirmed that they can enter the lungs through the liver and cause pulmonary fibrosis. Our study showed that these genes are positively associated with the development of IPF.

Four bacterial taxa, namely, FamilyXIII, Oscillibacter, Ruminococcusgnavus and Selenomonadales, are involved primarily in the SCFA metabolic pathway and thus protect against IPF. FamilyXIII is involved in the metabolism and regulation of SCFAs, thereby protecting the intestines and lungs.^[41]^ Oscillibacter can degrade carbohydrates, and an increase in its abundance promotes an increase in butyric acid levels.^[42]^ Ruminococcus gongavus could produce butyric acid, which can alleviate inflammatory and immune responses in the gut, thus maintaining the stability of the intestinal mucosal barrier. It can also be involved in the body energy metabolism.^[43]^ Selenomonadales can take advantage of intestinal carbohydrates or amino acids to produce acetic acid and butyric acid, and these SCFAs can alleviate intestinal inflammation.^[44,45]^ Veillonella was also linked to IPF in our study. However, studies on how these floras affect IPF are rare. In the future, additional studies on the mechanisms of the intestinal flora should be conducted to help us better develop personalized probiotic treatment recommendations for IPF patients.

## 5. Strengths and weaknesses

Notably, our study several advantages. First, we used the analysis of MR to investigate the causal relationship between GM and IPF, avoiding the influence of confounding factors on the study results and providing a particular GM for subsequent studies. Second, our study offers new perspective regarding the relationship between the GM and IPF at the genetic and hereditary levels; therefore, the risk of IPF in patients with disorders of the intestinal flora should be closely monitored to guide the prevention of IPF.

Our study has some limitations. First, although the majority of subjects in the Mibiogen database originated from European populations, its population stratification is also unclear, and the results might not be fully generalized to individuals of non-European ancestry. Second, the data from the Mibiogen database that we used were aggregated; therefore, we were unable to analyze the data in multiple subgroups, for example, to differentiate between sex subgroups of IPF patients. Therefore, we cannot come to a more convincing conclusion. Third, to obtain more comprehensive GWAS results, we used a significance threshold of *P* < 1 × 10^-5^. This adjustment may cause slight deviations in the results. Fourth, the results of this manuscript were not corrected with multiple testing. Fifth, our study did not involve the study of intrinsic mechanisms, nor did we use clinical data to validate the findings. Therefore, more research is required in the future to investigate the pathways of action and therapeutic targets involved in the association between GM and IPF.

## 6. Conclusion

Overall, we demonstrated the potential causal effect between GM and IPF using the method of MR analysis. These results will help us to combine the role of GM to develop a reasonable therapeutic plan and enrich the standardized treatment of IPF. In addition, our study also offered clues to the immune mechanism and genome sequencing studies of GM on lung diseases.

## Acknowledgments

We would like to acknowledge these 2 databases that are available and publicly accessible and available. Summary statistics for exposed GWAS are available from the MiBioGen(www.mibiogen.org), and GWAS data on outcomes are available from the FinnGen database(https://r9.risteys.finngen.fi/).

## Author contributions

**Conceptualization:** Shiqin Fan, Baorui Xue.

**Formal analysis:** Shiqin Fan.

**Funding acquisition:** Jing Ma.

**Investigation:** Baorui Xue.

**Methodology:** Shiqin Fan.

**Project administration:** Jing Ma.

**Software:** Shiqin Fan, Baorui Xue.

**Supervision:** Shiqin Fan.

**Visualization:** Shiqin Fan, Baorui Xue.

**Writing – review & editing:** Shiqin Fan.

## Supplementary Material


